# Need for Objective Task-Based Evaluation of Image Segmentation Algorithms for Quantitative PET: A Study with ACRIN 6668/RTOG 0235 Multicenter Clinical Trial Data

**DOI:** 10.2967/jnumed.123.266018

**Published:** 2024-03

**Authors:** Ziping Liu, Joyce C. Mhlanga, Huitian Xia, Barry A. Siegel, Abhinav K. Jha

**Affiliations:** 1 Department of Biomedical Engineering, Washington University, St. Louis, Missouri;; 2 Mallinckrodt Institute of Radiology, Washington University School of Medicine, St. Louis, Missouri; and; 3 Alvin J. Siteman Cancer Center, Washington University School of Medicine, St. Louis, Missouri

**Keywords:** task-based evaluation, multicenter clinical trial, segmentation, quantitative imaging, deep learning, artificial intelligence

## Abstract

Reliable performance of PET segmentation algorithms on clinically relevant tasks is required for their clinical translation. However, these algorithms are typically evaluated using figures of merit (FoMs) that are not explicitly designed to correlate with clinical task performance. Such FoMs include the Dice similarity coefficient (DSC), the Jaccard similarity coefficient (JSC), and the Hausdorff distance (HD). The objective of this study was to investigate whether evaluating PET segmentation algorithms using these task-agnostic FoMs yields interpretations consistent with evaluation on clinically relevant quantitative tasks. **Methods:** We conducted a retrospective study to assess the concordance in the evaluation of segmentation algorithms using the DSC, JSC, and HD and on the tasks of estimating the metabolic tumor volume (MTV) and total lesion glycolysis (TLG) of primary tumors from PET images of patients with non–small cell lung cancer. The PET images were collected from the American College of Radiology Imaging Network 6668/Radiation Therapy Oncology Group 0235 multicenter clinical trial data. The study was conducted in 2 contexts: (1) evaluating conventional segmentation algorithms, namely those based on thresholding (SUV_max_40% and SUV_max_50%), boundary detection (Snakes), and stochastic modeling (Markov random field–Gaussian mixture model); (2) evaluating the impact of network depth and loss function on the performance of a state-of-the-art U-net–based segmentation algorithm. **Results:** Evaluation of conventional segmentation algorithms based on the DSC, JSC, and HD showed that SUV_max_40% significantly outperformed SUV_max_50%. However, SUV_max_40% yielded lower accuracy on the tasks of estimating MTV and TLG, with a 51% and 54% increase, respectively, in the ensemble normalized bias. Similarly, the Markov random field–Gaussian mixture model significantly outperformed Snakes on the basis of the task-agnostic FoMs but yielded a 24% increased bias in estimated MTV. For the U-net–based algorithm, our evaluation showed that although the network depth did not significantly alter the DSC, JSC, and HD values, a deeper network yielded substantially higher accuracy in the estimated MTV and TLG, with a decreased bias of 91% and 87%, respectively. Additionally, whereas there was no significant difference in the DSC, JSC, and HD values for different loss functions, up to a 73% and 58% difference in the bias of the estimated MTV and TLG, respectively, existed. **Conclusion:** Evaluation of PET segmentation algorithms using task-agnostic FoMs could yield findings discordant with evaluation on clinically relevant quantitative tasks. This study emphasizes the need for objective task-based evaluation of image segmentation algorithms for quantitative PET.

## Visual Abstract

**Figure:**
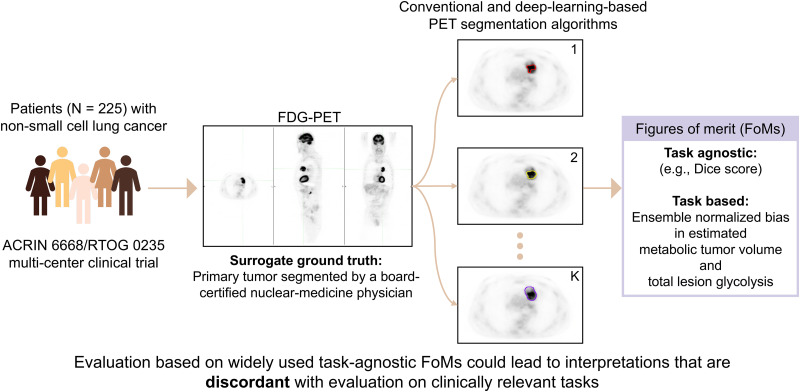

PET-derived quantitative metrics, such as tumor volumetric and radiomic features, are showing strong promise in multiple oncologic applications (1–3). Reliable quantification of these features requires accurate segmentation of tumors on the PET images. To address this need, multiple computer-aided image segmentation algorithms have been developed (4), including those based on deep learning (DL) (5–8). Clinical translation of these image segmentation algorithms requires objectively evaluating them with patient data.

Medical images are acquired for specified clinical tasks; thus, it is important that the performance of imaging and image-analysis algorithms be objectively assessed on those tasks. In this context, strategies have been proposed for task-based assessment of image quality (9–12). However, imaging algorithms, including those based on DL, are often evaluated using figures of merit (FoMs) that are not explicitly designed to measure clinical task performance (11). Recent studies conducted specifically in the context of evaluating image-denoising algorithms showed that task-agnostic FoMs may yield interpretations that are inconsistent with evaluation on clinical tasks (13–17). For example, in Yu et al. (17) a DL-based denoising algorithm for myocardial perfusion SPECT indicated significantly superior performance based on a structural similarity index measure and mean squared error but did not yield any improved performance on the clinical task of detecting myocardial perfusion defects.

Similar to image denoising, algorithms for image segmentation are almost always evaluated using FoMs that are not explicitly designed to quantify clinical task performance (5,18–21). These FoMs, including the Dice similarity coefficient (DSC), the Jaccard similarity coefficient (JSC), and the Hausdorff distance (HD) (4), quantify some measure of similarity between the predicted segmentation and a reference standard such as manual delineation. For example, the DSC measures spatial overlap between the predicted segmentation and reference standard. A higher value of DSC is typically used to infer more accurate performance. However, it is unclear how these task-agnostic FoMs correlate with performance on clinically relevant tasks.

Our objective was to investigate whether evaluating PET segmentation algorithms using task-agnostic FoMs leads to interpretations that are consistent with evaluation based on clinical task performance. Performing this investigation with patient data in a multicenter setting is highly desirable because such a study offers the ability to model variabilities in both patient population and clinical scanner configurations. Toward this goal, we conducted a retrospective study using data from the American College of Radiology Imaging Network (ACRIN) 6668/Radiation Therapy Oncology Group (RTOG) 0235 multicenter clinical trial (22,23). In this trial, patients with stage IIB/III non–small cell lung cancer were imaged with ^18^F-FDG PET/CT studies. In the study of non–small cell lung cancer, there is a strong interest in investigating whether early changes in tumor metabolism can help predict therapy response (24). Although most studies have focused on SUV-based metrics, the findings have been inconsistent (24,25), motivating the need for new and improved metrics. In this context, metabolic tumor volume (MTV) and total lesion glycolysis (TLG) are showing strong promise as prognostic biomarkers in multiple studies (3,26,27). As introduced above, computing these features requires tumor segmentation. Thus, our study was designed to assess the concordance in evaluating various image segmentation algorithms using task-agnostic metrics (DSC, JSC, and HD) versus on the clinically relevant tasks of estimating the MTV and TLG. Initial results of this study were presented in brief previously (28); here, we provide a detailed description of the methods and study design, provide new findings, and conduct comprehensive analyses of the results.

## MATERIALS AND METHODS

### Study Population

This retrospective study of existing data was approved by the institutional review board, which waived the requirement to obtain informed consent. Deidentified ^18^F-FDG PET/CT images of 225 patients with inoperable stage IIB/III locally advanced non–small cell lung cancer were collected from the ACRIN 6668/RTOG 0235 multicenter clinical trial (22,23). The images were collected from The Cancer Imaging Archive database (29). Baseline PET/CT scans were acquired before curative-intent chemoradiotherapy for each patient. Demographics and clinical characteristics of the patient population are summarized in Supplemental Table 1 (supplemental materials are available at http://jnm.snmjournals.org). A standardized imaging protocol was detailed by Machtay et al. (23). Briefly, an ^18^F-FDG dose ranging from 370 to 740 MBq was administered, with image acquisition beginning 50–70 min later and including the body from the upper–mid neck to proximal femurs. The PET images were acquired from 12 ACRIN-qualified clinical scanners (30), including GE Healthcare Discovery LS/ST/STE/RX, GE Healthcare Advance, Philips Allegro/Guardian, and CTI PET Systems (marketed as Siemens scanners): models 1023/1024/1062/1080/1094. The image reconstruction procedure compensated for attenuation, scatter, randoms, normalization, decay, and dead time. Details of the reconstruction protocol for each PET scanner are provided in Supplemental Table 2.

### Data Curation

**FIGURE 1.**
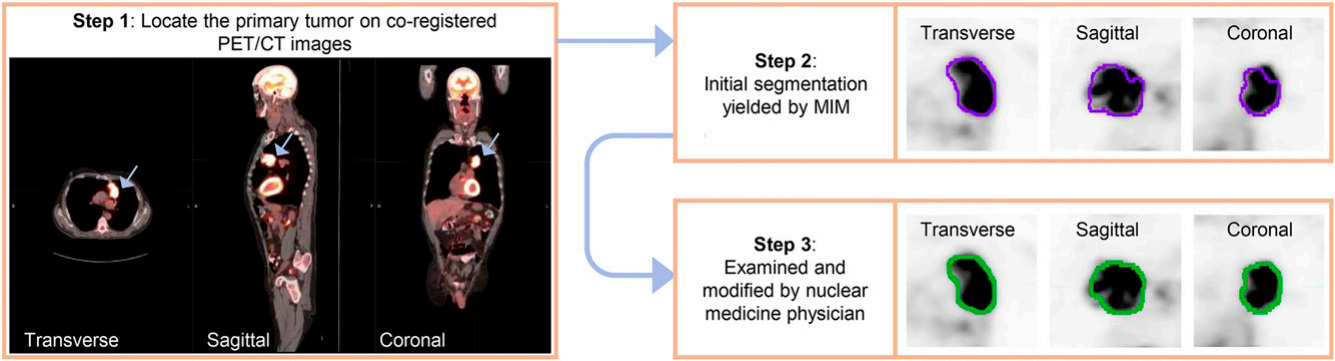
Workflow to obtain manual segmentation of primary tumor (arrow) for each patient. MIM = MIM Encore 6.9.3.

Evaluation of PET segmentation algorithms required knowledge of true tumor boundaries or a surrogate for ground truth, such as tumor delineations performed by an expert human reader. For this purpose, a board-certified nuclear medicine physician with more than 10 y of experience reading PET scans was tasked with defining the boundary of the primary tumor for each patient (Fig. 1). The physician was instructed to locate the primary tumor by carefully reviewing the coregistered PET/CT images along coronal, sagittal, and transverse planes and then using an edge-detection tool (MIM Encore 6.9.3; MIM Software Inc.) to obtain an initial boundary of the primary tumor. The physician was informed explicitly about potential errors in this initial boundary and was thus advised to review this boundary carefully and make any modifications as needed. The task of segmenting the tumors in the whole dataset was split into multiple sessions to avoid reader fatigue. At the end of this process, we had expert-defined segmentations for the primary tumors in the 225 PET scans in our dataset.

### Consideration of Conventional Computer-Aided Image Segmentation Algorithms

Conventional computer-aided PET segmentation algorithms are typically categorized into those based on thresholding, boundary detection, and stochastic modeling (4). We selected the algorithms of SUV_max_ thresholding (SUV_max_40% and SUV_max_50%) (31), Snakes (32), and Markov random field-Gaussian mixture model (MRF-GMM) (33) from each of those categories, respectively. A detailed description of these algorithms is provided in the supplemental materials (31–33).

### Consideration of DL-Based Image Segmentation Algorithm

We next considered the evaluation of a state-of-the-art U-net–based algorithm (5,8,34,35). A detailed description of the network architecture is provided in Supplemental Figure 1. When DL-based algorithms are developed and evaluated, common factors known to impact the performance include the choice of network depth (36), network width (37), loss function (38), and data preprocessing and augmentation strategies. In this study, we focused on investigating whether evaluating the impact of network depth and loss function using the task-agnostic FoMs yields inferences that are consistent with evaluation on the tasks of estimating MTV and TLG.

#### Network Training

The U-net–based algorithm was implemented to segment the primary tumor on 3-dimensional PET images on a per-slice basis. During training, 2-dimensional PET images of 180 patients with the corresponding surrogate ground truth (tumor delineations performed by the physician) were input into the U-net–based algorithm. The network was trained to minimize a loss function between the true and predicted segmentations using the Adam optimization method (39). The loss function will be specified in each experiment described below. Network hyperparameters, including parameters of activation function and dropout probability, were optimized via 5-fold cross-validation on the training dataset. The final optimized U-net–based algorithm was then evaluated on the remaining independent 45 patients from the same cohort. There was no overlap between the training and test sets.

#### Configuring the U-Net–Based Algorithm with Different Network Depths

We varied the network depth by setting the number of paired blocks of convolutional layers (supplemental materials) in the encoder and decoder to 2, 3, 4, and 5. The detailed network architecture that consisted of 2 paired blocks is provided in Supplemental Table 3. For each choice of depth, the network was trained to minimize a binary cross-entropy (BCE) loss between the true and predicted segmentations, denoted by $s^{t}$ and $s^{p}$, respectively. The number of voxels in the PET image is denoted by *N*. The BCE loss is given by

$$\begin{array}{c} L_{\text{BCE}}\left(s^{t},\;s^{p}\right)=-\frac{1}{N}\sum_{n=1}^{N}\left[s_{n}^{t}\log\;s_{n}^{p}+\left(1-s_{n}^{t}\right)\log\left(1-s_{n}^{p}\right)\right].\; \end{array}$$ Eq. 1

The network with each depth choice was independently trained and cross-validated on the training dataset. After training, each network was evaluated on the 45 test patients.

#### Configuring the U-Net–Based Algorithm with Different Loss Functions

A commonly used loss function in DL-based segmentation algorithms is the combined Dice and BCE loss, which leverages the flexibility of Dice loss for handling class-imbalance problems and the use of BCE loss for curve smoothing (36). In this loss function, the weight of BCE loss is controlled by a hyperparameter, denoted by λ. We investigated whether evaluating the impact of different values of λ on the performance of the U-net–based algorithm using the task-agnostic and task-based FoMs yields consistent interpretations.

The Dice loss is denoted by $L_{\text{Dice}}$, such that

$$\begin{array}{c} L_{\text{Dice}}\left(s^{t},\;s^{p}\right)=1-\frac{2\times \sum_{n=1}^{N}s_{n}^{t}s_{n}^{p}}{\sum_{n=1}^{N}s_{n}^{t}+\sum_{n=1}^{N}s_{n}^{p}}.\; \end{array}$$ Eq. 2

The combined Dice and BCE losses are defined as

$$\begin{array}{c} L_{\text{comb}}\left(s^{t},\;s^{p}\right)=\text{λ}L_{\text{BCE}}+\left(1-\text{λ}\right)L_{\text{Dice}}, \end{array}$$ Eq. 3

where the term $L_{\text{BCE}}$ is defined in Equation 1. In this experiment, we considered 6 different values of λ ranging from 0 to 1. We fixed the depth of the network by considering 3 paired blocks of convolutional layers in the encoder and decoder. For each value of λ, the network was independently trained and cross-validated on the same training dataset. Each trained network was then evaluated on the 45 test patients.

### Evaluation FoMs

#### Task-Agnostic FoMs

The widely used task-agnostic FoMs of DSC, JSC, and HD were used in this study. The DSC and JSC, as defined in Taha and Hanbury (40), measure the spatial overlap between the true and predicted segmentations. The values of both DSC and JSC lie between 0 and 1, and a higher value implies a more accurate performance. The HD quantifies the shape similarity between the true and predicted segmentations, and a lower value implies a more accurate performance. The values of DSC, JSC, and HD are reported as mean and 95% CI. Paired sample *t*-tests were performed to assess whether significant differences exist.

#### Task-Based FoMs

An essential criterion in validating algorithms to extract quantitative imaging metrics such as MTV and TLG is that the measurements obtained with the algorithm are accurate (41,42), because an algorithm that yields biased measurements would not correctly reflect the underlying pathophysiology. In a population, the bias can often vary on the basis of the true value and thus should be quantified over the entire measurable range of values to provide a more complete measure of accuracy (43). Ensemble normalized bias, defined as the bias averaged over the distribution of true values, helps address this issue and provides a summarized FoM for accuracy (44,45). This FoM was thus used in this study. Detailed definitions of the ensemble normalized bias are provided in the supplemental materials (41,42,44,45).

## RESULTS

### Evaluation of Conventional Computer-Aided Algorithms

**FIGURE 2.**
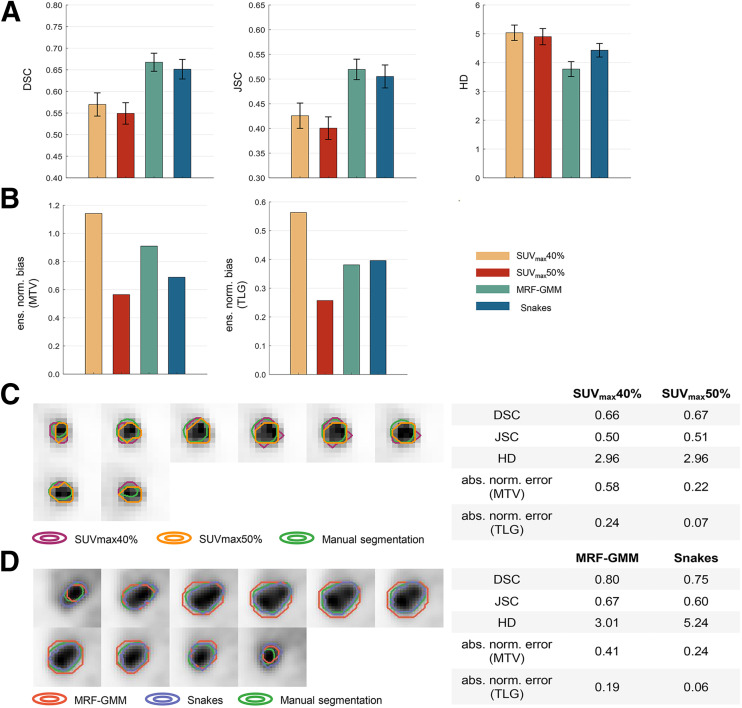
Quantitative assessment of concordance in evaluation of considered conventional PET segmentation algorithms using task-agnostic FoMs of DSC, JSC, and HD (A) and on tasks of estimating MTV and TLG of primary tumor (B). Comparisons of segmentations yielded by SUV_max_40% vs. SUV_max_50% (C) and MRF-GMM vs. Snakes (D) were provided for 2 representative patients. ens. norm. = ensemble normalized; abs. norm. = absolute normalized.

Figures 2A and 2B present the quantitative assessment of conventional computer-aided segmentation algorithms over the 225 patients using the task-agnostic and task-based FoMs. On the basis of DSC and JSC, SUV_max_40% significantly outperformed SUV_max_50% (*P* < 0.05). However, we observed that SUV_max_40% yielded increased ensemble normalized bias in the estimated MTV and TLG of 51% and 54%, respectively, indicating a much less accurate performance on the clinically relevant quantitative tasks. Similarly, the MRF-GMM significantly outperformed Snakes on the basis of the DSC, JSC, and HD (*P* < 0.05) but revealed a 24% increased ensemble normalized bias in the estimated MTV.

Figure 2C shows the visual comparison of segmentations yielded by SUV_max_40% versus SUV_max_50% for a representative patient. We observed that both algorithms yielded very similar DSC, JSC, and HD values. However, SUV_max_40% yielded substantially higher absolute normalized error (aNE) in the estimated MTV and TLG. For another representative patient shown in Figure 2D, the MRF-GMM yielded higher DSC and JSC and lower HD values. However, this algorithm yielded less accurate estimates of MTV and TLG, as indicated by the higher aNEs.

### Evaluating the U-Net–Based Algorithm

#### Impact of Network Depth Choice

**FIGURE 3.**
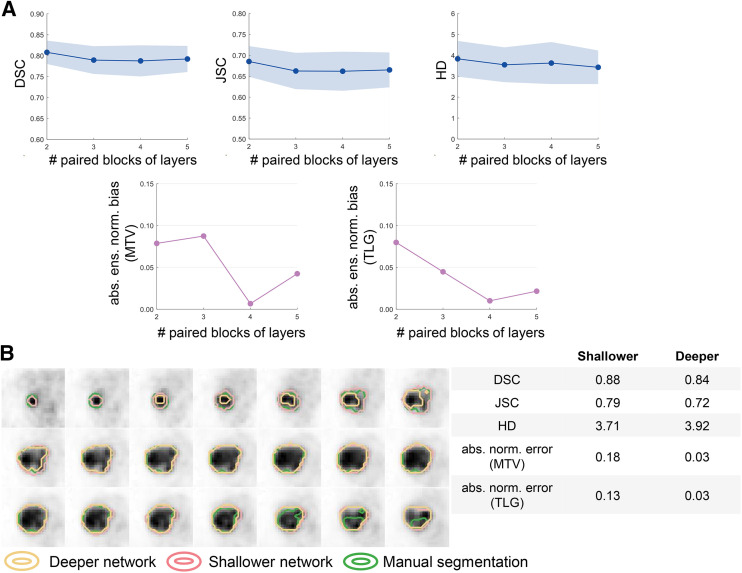
(A) Quantitative assessment of concordance between task-agnostic and task-based FoMs in evaluating impact of varying network depth on performance of U-net–based algorithm. (B) Comparison of segmentations yielded by deeper and shallower network for 1 representative test patient. abs. ens. norm. = absolute ensemble normalized; abs. norm. = absolute normalized.

Figure 3A shows the impact of varying network depth on the performance of the U-net–based algorithm, as evaluated using both the task-agnostic and the task-based FoMs on the 45 test patients. No significant difference was detected among any of the considered network depths on the basis of the DSC, JSC, and HD (*P* < 0.05). However, deeper networks yielded more accurate performance on the tasks of estimating MTV and TLG. Particularly, compared with the shallower network with 2 paired blocks of convolutional layers, the deeper network with 4 paired blocks yielded substantially lower absolute ensemble normalized bias in the estimated MTV and TLG, with a decrease of 91% and 87%, respectively. Segmentations of the shallower and deeper networks are shown for 1 representative test patient in Figure 3B. We observed that the deeper network yielded lower DSC and JSC and higher HD values but actually outperformed the shallower network on the tasks of estimating the MTV and TLG.

#### Impact of Loss Function Choice

**FIGURE 4.**
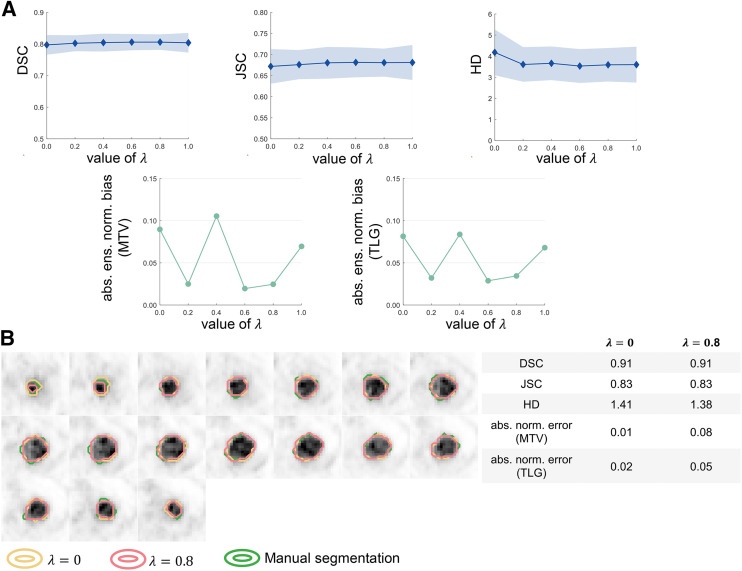
(A) Quantitative assessment of concordance between task-agnostic and task-based FoMs in evaluating impact of loss function on performance of U-net–based algorithm. (B) Comparison of segmentations yielded by U-net–based algorithm configured with 2 loss functions for 1 representative test patient. abs. ens. norm. = absolute ensemble normalized; abs. norm. = absolute normalized.

Figure 4A shows the assessment of concordance between task-agnostic versus task-based FoMs in evaluating the impact of varying loss functions on the performance of the U-net–based algorithm. On the basis of the DSC, JSC, and HD, there was no significant difference among any values of the hyperparameter, λ. However, we observed substantial variations in the tasks of estimating MTV and TLG, with up to a 73% and 58% difference between the highest and lowest ensemble normalized bias in the estimated MTV and TLG, respectively. Figure 4B compares the segmentations obtained with a λ of 0 versus a λ of 0.8 for a representative test patient. For this patient, whereas the values of DSC, JSC, and HD were similar, a λ of 0 yielded lower aNEs in the estimated MTV and TLG.

## DISCUSSION

Reliable performance on clinically relevant tasks is crucial for clinical translation of image segmentation algorithms. A key task for which image segmentation is often conducted in oncologic PET is quantifying features such as MTV and TLG. However, these segmentation algorithms are almost always evaluated using FoMs that are not explicitly designed to measure clinical task performance. In this study, we investigated whether evaluating PET segmentation algorithms with the widely used task-agnostic FoMs leads to interpretations that are consistent with evaluation on clinically relevant quantitative tasks.

Results from Figure 2 indicate that evaluation of conventional computer-aided PET segmentation algorithms based on task-agnostic FoMs of DSC, JSC, and HD could yield discordant interpretations compared with evaluation on the tasks of estimating MTV and TLG of the primary tumor. When evaluating the SUV_max_ thresholding algorithm, initial inspection based on the task-agnostic FoMs implied that the intensity threshold of 40% SUV_max_ yielded a significantly superior performance. However, further investigation showed that SUV_max_50% provided substantially more accurate performance on estimating MTV and TLG. This discordance was also observed when comparing the MRF-GMM and Snake algorithms. Thus, these results demonstrate the limited ability of the DSC, JSC, and HD to evaluate image segmentation algorithms on clinically relevant tasks.

The limitation in task-agnostic FoMs was again observed in evaluating the impact of network depth and loss function on the performance of a state-of-the-art U-net–based image segmentation algorithm. In Figure 3, we observed initially that the deeper networks yielded DSC, JSC, and HD values statistically similar to those in the shallower networks. Considering the requirement for computational resources when training DL-based algorithms, this may motivate the deployment of shallower networks in clinical studies. However, our task-based evaluation showed that a deeper network yielded substantially higher accuracy in the estimated MTV and TLG. Similarly, we observed from Figure 4 that based on the task-agnostic FoMs, the performance of the U-net–based algorithm was insensitive to the choice of λ (the hyperparameter controlling the weight of BCE loss in the cost function). However, differences up to 73% and 58% could exist between the highest and lowest ensemble normalized bias in the estimated MTV and TLG, respectively.

**FIGURE 5.**
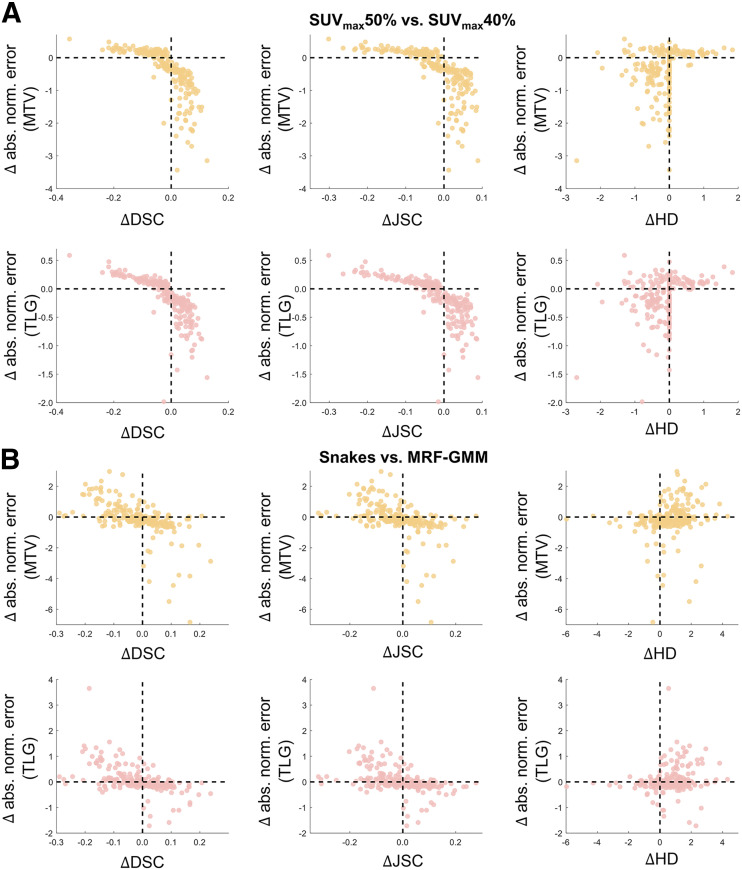
Quantitative assessment of concordance between interpretations obtained with task-agnostic vs. task-based FoMs on per-patient basis for considered computer-aided PET segmentation algorithms. Each point in scatter diagram represents individual patient. Horizontal position of each point indicates difference in DSC, JSC, and HD between SUV_max_50% vs. SUV_max_40% (A) and MRF-GMM vs. Snakes (B). Similarly, vertical position indicates difference in aNEs in estimated MTV and TLG. abs. norm. = absolute normalized.

**FIGURE 6.**
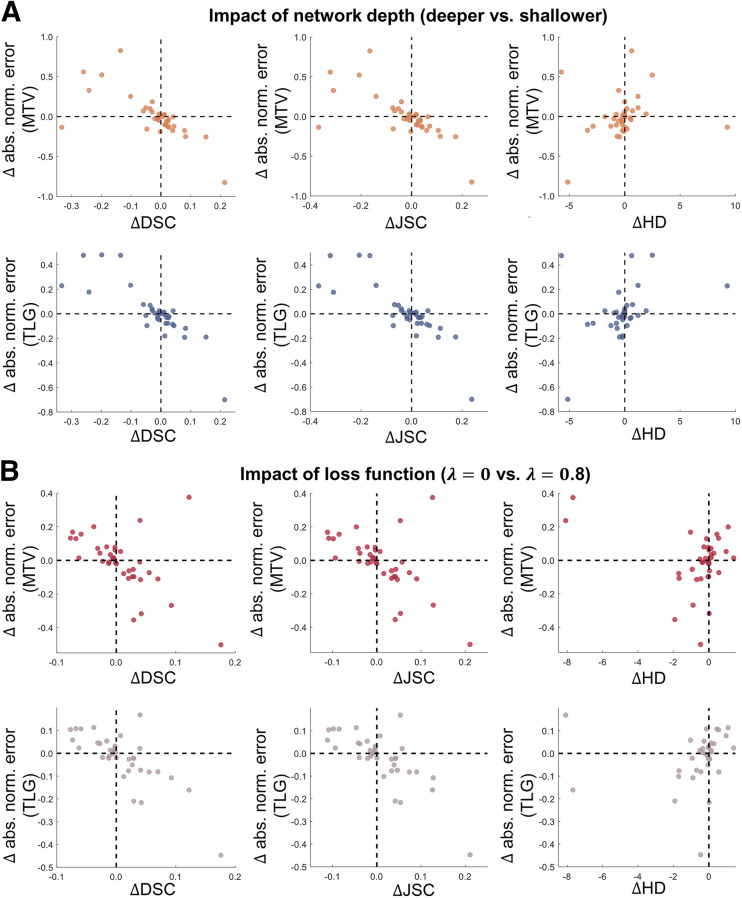
Quantitative assessment of concordance between interpretations obtained with task-agnostic vs. task-based FoMs on per-patient basis when evaluating impact of network depth (A) and loss function (B) on performance of U-net–based algorithm. abs. norm. = absolute normalized.

To gain further insights into the observed discordance between task-agnostic and task-based FoMs, we performed secondary analyses on a per-patient basis. In Figure 5A, for each of the 225 patients, we first calculated the difference (Δ) in DSC, JSC, and HD between SUV_max_50% and SUV_max_40% (e.g., $\text{ΔDSC}={\text{DSC[SUV}}_{\text{max}}50\text{\%]}\;-{\;\text{[DSC[SUV}}_{\text{max}}40\text{\%]}$). Next, we obtained the difference in the aNE (supplemental materials; Eq. 2) in the estimated MTV and TLG (e.g., $\text{MTV}\;\text{ΔaNE}=\text{MTV}\;\text{aNE}[{\text{SUV}}_{\text{max}}50\%]-\text{MTV}\;\text{aNE}[{\text{SUV}}_{\text{max}}40\%]$). We then studied the relationship between ΔDSC (and ΔJSC and ΔHD) versus ΔMTV aNE (and ΔTLG aNE) via scatter diagrams. For 36 patients, a negative value of ΔDSC/ΔJSC was observed, implying that SUV_max_50% was inferior to SUV_max_40%. However, for these patients, SUV_max_50% actually yielded better estimates of MTV, as indicated by the lower aNEs. Similarly, it was observed that interpretations obtained with ΔHD could be discordant with those based on ΔMTV aNE/ΔTLG aNE. Additionally, even for minor changes in DSC, JSC, and HD (i.e., ΔDSC/ΔJSC/ΔHD≈0; close to the vertical dashed line in the scatter diagram), we observed substantial variations in the ΔMTV aNE/ΔTLG aNE values. This indicates that these task-agnostic FoMs could be insensitive to even dramatic changes in quantitative task performance. This trend was again observed when comparing MRF-GMM versus Snakes (Fig. 5B) and evaluating the impact of network depth and loss function on the performance of the U-net–based algorithm (Fig. 6).

The findings of this study are not meant to suggest that the task-agnostic metrics, including the DSC, JSC, and HD, are not helpful. In fact, initial development of segmentation algorithms may not be associated with a specific task, and thus, task-agnostic FoMs are valuable for assessing the promise of these algorithms. However, for clinical application, it is important to further assess the performance of these algorithms on clinical tasks for which imaging is performed, as also emphasized in the best practices for evaluation of artificial intelligence algorithms for nuclear medicine (RELAINCE guidelines) (44). Results from our study further confirm the need for this task-based evaluation.

Our task-based evaluation focused on assessing the accuracy of image segmentation algorithms in quantifying features from PET images. In clinical studies, other criteria to evaluate the quantification performance could include precision, when repeatability or reproducibility are required for clinical decision-making. When the segmentation is required for radiotherapy planning, the relevant criterion is therapeutic efficacy—for example, the task of improving the probability of tumor control while minimizing the chances of normal-tissue complications. For this task, Barrett et al. proposed the use of an area under the therapy operating characteristic curve (46) for evaluating the segmentation algorithms. In all of these evaluation studies, clinicians (radiologists, nuclear medicine physicians, and disease specialists) have a crucial role in defining the clinically most relevant task and corresponding FoMs for the evaluation of image segmentation algorithms (11).

Evaluating PET segmentation algorithms on quantification tasks required knowledge of true quantitative values of interest. However, such ground truth is often unavailable in clinical studies. To circumvent this challenge, we considered quantitative values obtained using expert human-reader–defined manual delineations as surrogate ground truth. However, we recognize that this surrogate may be erroneous. To address the issue of a lack of ground truth in task-based evaluation of quantitative imaging algorithms, no-gold-standard evaluation techniques have been developed (47–50). These techniques have demonstrated promise in evaluating PET segmentation algorithms on clinically relevant quantitative tasks (51–53). As these techniques are validated further, they could provide a mechanism to perform objective task-based evaluation of segmentation algorithms with patient data. The findings from this study motivate further development and validation of these no-gold-standard evaluation techniques.

Other limitations of this study include the fact that the PET scanners used in the ACRIN 6668/RTOG 0235 multicenter clinical trial were relatively old and did not have time-of-flight capability. Thus, these scanners could yield substantially lower effective sensitivity compared with modern PET scanners. Conducting the proposed study with newer-generation scanners could provide further insights into the potential discordance between task-agnostic and task-based FoMs with more modern technologies. Additionally, the U-net–based algorithm was trained to segment tumors on a per-slice basis. As shown by Leung et al. (5), this strategy helped alleviate the requirement for large amounts of training data and the demand for computational resources. Results from this study motivate expanding the evaluation of 3-dimensional fully automated DL-based algorithms.

As a final remark, the purpose of this study was not to compare DL-based algorithms with conventional computer-aided algorithms. Although we observed that the considered U-net–based algorithm yielded substantially improved performance compared with conventional algorithms based on the task-agnostic and task-based metrics, this study does not intend to suggest that DL-based algorithms are preferable over conventional algorithms.

## CONCLUSION

Our retrospective analysis with the ACRIN 6668/RTOG 0235 multicenter clinical trial data shows that evaluation of PET segmentation algorithms based on widely used task-agnostic FoMs could lead to findings that are discordant with evaluation on clinically relevant quantitative tasks. The results emphasize the important need for objective task-based evaluation of image segmentation algorithms for quantitative PET.

## DISCLOSURE

This work was supported by the National Institute of Biomedical Imaging and Bioengineering through R01-EB031051, R01-EB031962, R56-EB028287, and R21-EB024647 (Trailblazer Award). No other potential conflict of interest relevant to this article was reported.

## Notes

KEY POINTS **QUESTION:** Are widely used metrics such as DSC, JSC, and HD sufficient to evaluate image segmentation algorithms for their clinical applications? **PERTINENT FINDINGS:** Our retrospective analysis with the ACRIN 6668/RTOG 0235 multicenter clinical trial data shows that evaluating PET segmentation algorithms on the basis of the DSC, JSC, and HD FoMs could lead to interpretations that are discordant with evaluation on the clinically relevant quantitative tasks of estimating the MTV and TLG of primary tumors in patients with non–small cell lung cancer. **IMPLICATIONS FOR PATIENT CARE:** Objective task-based evaluation of new and improved image segmentation algorithms is important for their clinical application.
